# Under the influence: environmental factors as modulators of neuroinflammation through the IL-10/IL-10R axis

**DOI:** 10.3389/fimmu.2023.1188750

**Published:** 2023-08-03

**Authors:** Eryn Bugbee, Angela A. Wang, Jennifer L. Gommerman

**Affiliations:** Department of Immunology, University of Toronto, Toronto, ON, Canada

**Keywords:** multiple sclerosis, central nervous system, experimental autoimmune encephalomyelitis, IL-10, IL-10R, environmental factors, B cells

## Abstract

The IL-10/IL-10 receptor (IL-10R) axis plays an important role in attenuating neuroinflammation in animal models of Multiple Sclerosis (MS) and increased IL-10 has been associated with a positive response to MS disease modifying therapy. Because environmental factors play an important role in MS susceptibility and disease course, identification of environmental factors that impact the IL-10/IL-10R axis has therapeutic potential. In this review, we provide historical and updated perspectives of how IL-10R signaling impacts neuroinflammation, discuss environmental factors and intestinal microbes with known impacts on the IL-10/IL-10R axis, and provide a hypothetical model for how B cells, via their production of IL-10, may be important in conveying environmental “information” to the inflamed central nervous system.

## 1 The IL-10/IL-10R axis

In the context of disease, IL-10 and its cognate receptor IL-10R have been implicated in mitigating autoreactive T cell responses. One such context is multiple sclerosis (MS), a chronic, inflammatory disease of the central nervous system (CNS) that affects over 2 million people worldwide (1). The disease exhibits heterogeneous clinical presentation and is characterized by the infiltration of lymphocytes into the brain and spinal cord, resulting in demyelination and axonal loss (2). The animal model of MS, Experimental Autoimmune Encephalomyelitis (EAE), has been pivotal to our understanding of how such autoreactive T cells are primed, infiltrate the CNS and set up an inflammatory milieu that promotes demyelinating lesions (3). Early studies showed that myelin-specific Th2 cells could inhibit EAE via their production of Th2-associated cytokines (4–6). Subsequent work using IL-10 knockout and transgenic overexpression revealed that IL-10 is a key regulatory cytokine required to regulate EAE (7–9). However, the use of therapeutic IL-10 administration has yielded inconsistent outcomes in both EAE and MS (10–13). To contextualize these data, it is important to understand the underlying mechanism of IL-10 mediated anti-inflammatory processes and environmental factors that can modulate levels or activity of IL-10.

### 1.1 Historical significance of the IL-10/IL-10R axis

In 1989, Fiorentino and colleagues discovered a cytokine produced by Th2 cells acting directly on Th1 cells to inhibit their function *in vitro* (14). At the time, they named the secreted factor “cytokine synthesis inhibitory factor (CSIF)”, but it is now widely known as interleukin-10 (IL-10) (15, 16). Since this discovery, many innate immune cells (macrophages, monocytes, dendritic cells (DCs), and neutrophils) and adaptive immune cells (CD4^+^/CD8^+^ T cells and B cells) have been identified as producers of IL-10 (17). Early evidence supported the concept that IL-10 has an inhibitory effect on T effector cells via direct and indirect mechanisms (16, 18, 19). For example, IL-10 was shown to prevent T cell proliferation and cytokine production in an indirect manner by hampering the maturation and T cell stimulation capabilities of DCs (20–22), or by downregulating MHC class II expression on monocytes (23–26). On the other hand, IL-10 was also found to act directly on CD4^+^ T cells by inducing their anergy (27), suppressing the expansion of pathogenic Th17 cells (28, 29) and promoting the regulatory activity of CD4^+^ Foxp3^+^ regulatory T cells (Tregs) (30, 31) and CD4^+^ T regulatory type 1 (T_R_1) cells (32).

### 1.2 IL-10 producing cells

While T cells and myeloid cells collectively constitute a major cellular source of IL-10 (16, 33, 34), B cells also restrict inflammation via IL-10 in the context of neuroinflammation (as well as other autoimmune settings). Early work by Fillatreau and Anderton found that mice with B cell specific IL-10 deficiency fail to recover from EAE, and restoring this population with an adoptive transfer of IL-10^+^ B cells leads to disease recovery (35). Further studies have shown that regulatory B cell populations including Bregs, plasma cells (PCs) and plasmablasts can all limit the severity of EAE in an IL-10 dependent manner (36–39). The underlying regulatory mechanisms of B cell derived IL-10 are still being explored but it has been shown using human peripheral blood mononuclear cells (PBMCs) that plasmablast-derived IL-10 can hinder the ability of DCs to generate autoreactive T cells (39). Alternatively, IL-10^+^ Bregs in a murine model of arthritis have been shown to contribute to the induction of FoxP3^+^ Tregs and suppression of Th1/Th17 cells *in vivo* (37). Related human studies have found that MS patient B cells exhibit deficient IL-10 production following *ex vivo* stimulation (40). Following anti-CD20 induced MS remission, B cells that reconstitute the periphery regain their ability to produce IL-10 (41). Thus, B cell derived IL-10 plays a key role in regulating autoimmune inflammation and may contribute to the mechanism of action of anti-CD20 therapy in MS.

### 1.3 Downstream signaling through IL-10/IL-10R

Il-10 signals through the IL-10 receptor (IL-10R), a hetero-tetramer consisting of two alpha and two beta subunits (15). While the IL-10Rβ subunit can bind to other members of the IL-10 super family including IL-22 and IL-26 (34), the IL-10Rα subunit is specific to IL-10 (15). IL-10Rα is expressed at a basal level on most hematopoietic cells. However, certain immune populations have higher expression levels of IL-10Rα, especially upon immune activation (15). For instance, antigen-presenting cells (APCs) and other myeloid cells such as microglia have been shown to express high levels of IL-10Rα from development onwards (15, 42, 43). Conversely, naive CD4^+^ T-cells have low levels of steady state IL-10Rα expression that increases upon TCR stimulation both on multiple T-cells subsets *in vivo* and *in vitro* (28, 29).

**Figure 1:**
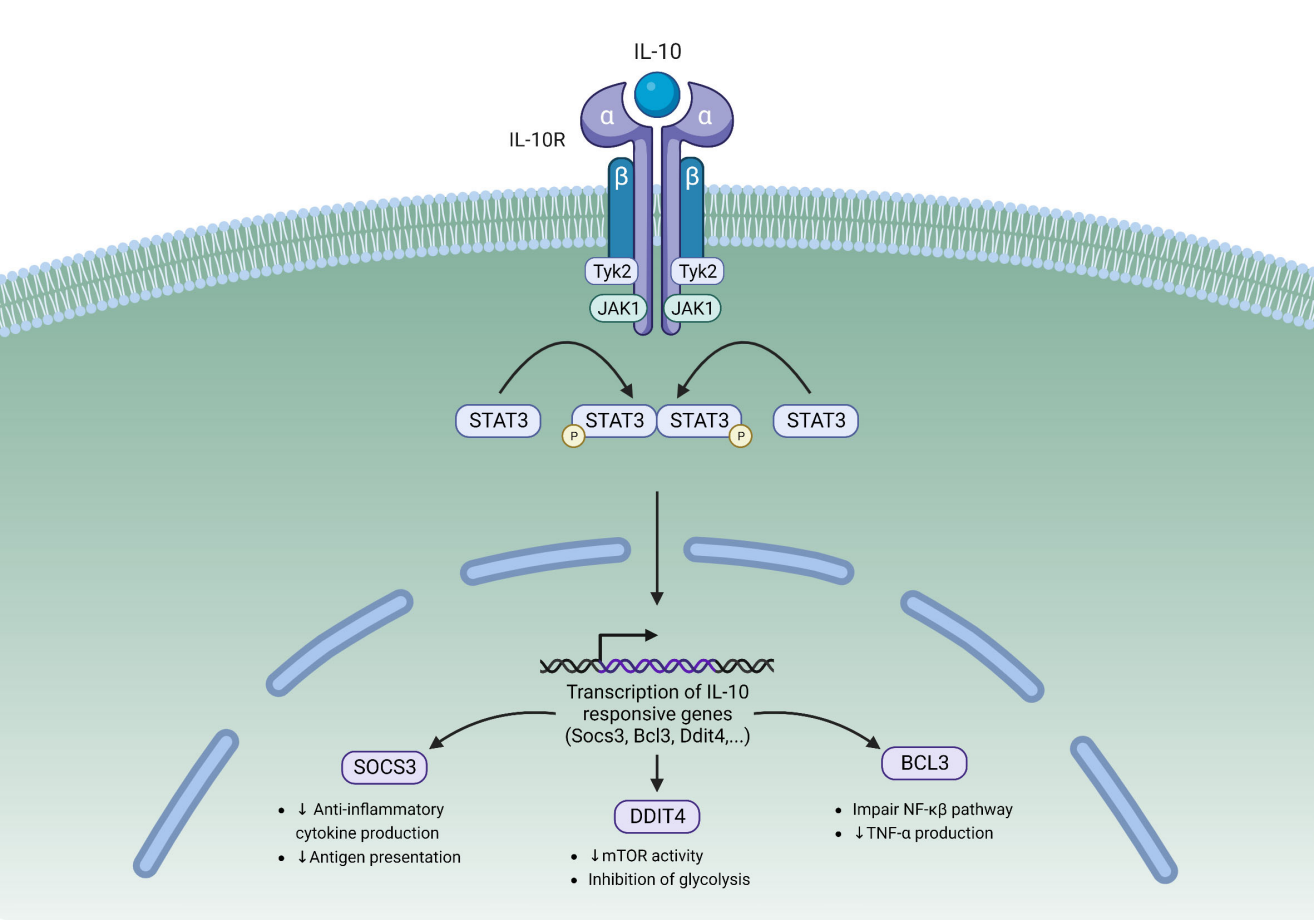
The IL-10/IL-10R Signaling pathway. IL-10 binding to its heterodimeric receptor leads to the phosphorylation of STAT3 by JAK1 and Tyk2. Upon phosphorylation, STAT3 translocates to the nucleus where it binds to STAT3-binding elements and activates the transcription of target genes. STAT3 is responsible for activating the transcription of several IL-10 responsive genes including SOCS3, Bcl3, and Ddit4. Tyk2, Tyrosine kinase 2; JAK1, Janus kinase 1; STAT3, Signal transducer and activator of transcription 3; SOCS3, Suppressor of cytokine signaling 3; DDIT4, DNA damage-inducible transcript 4.

Upon IL-10 binding, a cascade of intracellular signaling events occurs (**Figure 1**). This leads to the activation of tyrosine kinases Jak1 and Tyk2, which then reciprocally phosphorylate tyrosine residues of the IL-10Rα (15, 44). Receptor phosphorylation leads to the recruitment and phosphorylation of signal transducer and activator of transcription 3 (STAT3), which is then activated and subsequently translocates to the nucleus (45). In the nucleus STAT3 binds to STAT3-binding elements and activates the transcription of target genes, one of which is Suppressor Of Cytokine Signaling 3 (SOCS3). SOCS3 inhibits the transcription of pro-inflammatory cytokines such as IL-6 and TNFα. SOCS3 also inhibits IL-10 transcription, resulting in a negative feedback loop downstream of IL-10R signaling (15). Other STAT3 target genes include Bcl3, a known inhibitor of the NF-κβ pathway that can suppress the production of pro-inflammatory cytokines (46), and Ddit4, an mTOR inhibitor that has been shown to decrease the inflammatory activities of macrophages (47).

### 1.4 Importance of IL-10R signaling during homeostasis – a focus on the gut

Much of the current understanding of the impact of IL-10R signaling has been elucidated in the context of gastrointestinal diseases such as inflammatory bowel disease (IBD), where the constant interaction between immune cells and the gut microbiota demands strict regulation within the local intestinal milieu. In humans, mutations to the *IL10RA* and *IL10RB* genes have both been strongly associated with infant colitis associated with defects in downregulation of proinflammatory cytokine secretion by monocytes (48–50). Others have also found that impaired IL-10R signaling in adult IBD patients is associated with increased T cell polarization towards a Th17 lineage, decreased IL-10-induced STAT3 phosphorylation, and increased pro-inflammatory cytokine expression in monocytes following *in vitro* stimulation (51–53).

In mice, deletion of IL-10Rβ results in spontaneous colitis (54) and deletion of IL-10Rα specifically in macrophages increases susceptibility to chemically induced colitis (55). In addition, IL-10R signaling in Foxp3^+^ Tregs is critical for suppressing pathogenic Th17 cells and IL-10R signaling in Th17 cells directly suppresses their expansion (29, 32). Beyond T cells, IL-10Rα deletion in monocytes/macrophages leads to an increase in IL-17 and IL-6 proinflammatory cytokine levels in serum, the production of nitric oxide (NO) and reactive oxygen species (ROS) by lamina propria macrophages, and an overall proinflammatory gene expression signature in intestinal macrophages (56, 57). Similarly, anti-IL-10Rα antibody blockade increases the expression of pro-inflammatory and STAT1-inducible genes such as *Cxcl9* and *Cxcl11* in colonic macrophages (58). Elimination of IL-10R in CD11c^+^ cells, including DCs, is associated with an amplified immune response to bacterial and fungal pathogens as well as allergens in the skin (55, 59, 60).

Taken together, IL-10R signaling has clear immunoregulatory roles in restraining inflammation in the gut – an environment that is constantly exposed to microbial antigens.

## 2 IL-10/IL-10R axis in MS and EAE: some paradoxes

The IL-10/IL-10R axis has been implicated as a key mechanism for constraining inflammation during MS/EAE. Several EAE studies have found that both global and cell specific IL-10 knockout leads to worsened disease, yet therapeutic administration of IL-10 has had mixed results in EAE and MS (10–13). In this section we explore the pro- and anti-inflammatory effects of IL-10 during MS/EAE and the contexts that separate potentially helpful versus harmful impacts of this cytokine.

### 2.1 Anti-inflammatory effects of IL-10/IL-10R signaling in EAE and MS

In EAE, IL-10 deficiency leads to increased disease incidence and severity (8), and mice with APCs that over-express IL-10 driven by a class II MHC promoter are strongly resistant to the development of EAE (7, 8). Moreover, several studies have found that serum IL-10 levels in MS patients are decreased prior to and during disease relapses, but are increased during remission (61–67). Profiling of CCR6^+^ myelin-reactive CD4^+^ T cells from MS patients also found that these cells had decreased IL-10 production in comparison to healthy control T cells (68). Furthermore, treatment with first-line disease-modifying therapies DMTs such as glatiramer acetate (GA) and interferon-beta (IFNβ) is associated with increased IL-10 production by PBMCs isolated from EAE mice and MS patients (61, 62, 69, 70). Of note, treatment with fingolimod, GA, and IFNβ also increases the proportion of IL-10 producing B cells in MS patients (71–73).

Several IL-10 producing cells have been implicated in the regulation of MS and EAE including regulatory T cells (Tregs) and B cells (Bregs) (30, 35, 38). A higher frequency of IL-10 producing Tregs in the CNS during EAE has been shown to correlate with disease recovery and depletion of these cells leads to an exacerbation of disease (74). Moreover, during EAE the loss of Breg derived IL-10 prevents disease recovery and leads to a Th1-dominant response (35). A link between IL-10 producing B cells and Foxp3^+^ Tregs has also been established during EAE. B cell deficient mice were shown to have lower levels of both IL-10 and Foxp3 expression in the spinal cord during EAE, suggesting that B cells may play a role in promoting Foxp3^+^ Treg accumulation in the CNS through an IL-10 dependent manner (75). Furthermore, plasma cells- the terminally-differentiated B cell typically associated with antibody production, have also been shown to contribute to protection against EAE through the production of IL-10 (38, 39).

Despite the clear role for IL-10 in limiting the severity of EAE (7, 8), and its association with reduced white matter lesions and an improved Expanded Disability Status Scale (EDSS) score in MS (63, 76), relatively less is known about the impact of its cognate receptor IL-10Rα in regulating neuroinflammation. CD4^+^ T cells derived from the blood of MS patients are relatively hyporesponsive to the immunosuppressive function of IL-10 *in vitro* compared to healthy controls, and this hyporesponsiveness is associated with impaired STAT3 phosphorylation (77), suggesting defects in IL-10Rα signaling. In addition, one allele of the IL-10Rα S138G polymorphism, which encodes for a loss-of-function allele for IL-10-induced STAT1 and STAT3 activation (78) is associated with MS disease susceptibility and severity in Tunisians (79), and two mutant alleles confers an increased risk for MS specifically in men who are normally less susceptible than women in developing relapsing-remitting MS (79). The same polymorphism has been linked to a higher risk of ulcerative colitis (80) and systemic lupus erythematosus (81, 82).

### 2.2 Pro-inflammatory effects of IL-10/IL-10R signaling in EAE and MS

While the bulk of research indicates that IL-10/IL-10R signaling contributes to the dampening of EAE/MS, some studies suggest otherwise. For example, IL-10 mRNA levels in serum are increased in MS patients in comparison to healthy controls (83–85), and in PBMCs *IL10* mRNA levels are increased 2 weeks post-MSrelapse but subsequently return back to baseline after 4 weeks (64). However, it is unclear whether these increased levels of IL-10 mRNA/protein are involved in promoting pro-inflammatory conditions or represent a counter-regulatory mechanism that is triggered by neuroinflammation.

In two separate studies examining MOG_35-55_ EAE, IL-10rα deletion specifically in T cells reduced disease severity (86, 87). Liu et al. found that T cell specific *IL10Ra* deletion led to increased proportions of Tregs during the early phase of disease and an overall decrease in T cell accumulation during the disease course in the CNS and secondary lymphoid tissue (86). Using competitive bone marrow chimeras, T effector cells expressing *IL10Ra* exhibited a survival advantage over *Il10Ra*-deficient T effectors cells (86). In addition, Yogev et al. found that although CD4^+^ T cells are a relatively minor source of IL-10, T cell-derived IL-10 worsens EAE by acting on Th1 cells (and Th17 cells to a lesser extent) to promote their survival and proliferation in the CNS (87).

These results may explain why investigations into the therapeutic delivery of IL-10 have yielded mixed findings (10–13). As different immune populations produce IL-10 at different time points during MS/EAE, therapeutic efficacy could be dictated by the dose, delivery method, and timing.

## 3 Environmental factors that influence IL-10/IL-10R during MS/EAE

A person’s sex, age, diet, exercise, prior infections, geographic location, antibiotic use, exposure to pollution and early life factors (breastfeeding, mode of delivery) can all influence the composition of one’s microbiome (88). As such, the microbiome is a window into environmental exposures and accordingly has been studied for its potential role as a risk factor for MS incidence and/or severity (89, 90). However, there are also other environmental factors that can exert a direct impact on the immune system and by extension potentially on MS pathogenesis, independent of the microbiome. For example EBV infection and Vitamin D have been shown to act directly on immune cells *in vitro*, altering their functionality. In this section, we review how environmental factors can impact the IL-10/IL-10R axis in MS and EAE via the microbiome (section 3.1-3.2) or potentially independent of the microbiome (section 3.3-3.4)

### 3.1 The intestinal microbiome

The intestinal microbiome has a profound impact on host immunity even at distal sites such as the CNS (90, 91). Several human studies have revealed differences in the composition of the microbiome comparing patients with MS and healthy controls, the most common alterations being *Akkermansia*, *Acinetobacter*, and *Parabacteriodes* taxa (92–95). Shifts in microbiome composition in MS patients have also been associated with changes in immunomodulatory metabolites (96). However, causal associations between microbiome alterations in disease susceptibility or severity are difficult to establish in the real world. To address this, EAE models involving colonization of germ free or antibiotic treated mice via fecal microbial transplant (FMT) can be used to gain fundamental understanding into causality (94, 95).

Evidence that host commensal microbial communities influence IL-10 levels and subsequently CNS autoimmunity was first derived from antibiotic treatment studies. In these studies, oral administration of an antibiotic cocktail protected mice against the onset and severity of EAE. This phenomenon was associated with significantly increased levels of IL-10 secretion from cells isolated from secondary lymphoid tissue, specifically, IL-10 producing Foxp3^+^ Tregs (97, 98). Subsequently, *Bacteriodes fragilis*, a commensal bacteria that produces polysaccharide A (PSA), was found to be responsible for protection against EAE by triggering the activation of IL-10^+^ Tregs through the Toll-like receptor 2 pathway (99–102). Indeed, mice treated with oral PSA that were lacking IL-10 had similar clinical disease as wild-type mice, indicating that PSA and Treg mediated protection against disease requires IL-10 (99). Other intestinal commensal microbes have been implicated for their disease altering properties in EAE. For example, colonization with *Prevotella histicola* reduces EAE severity and is associated with increased IL-10 production by DCs (103). Cekanaviciute and colleagues identified a reduction in the bacterial genera *Parabacteroides distasonis* in MS patients and showed that *P. distasonis* exposure increases the differentiation of IL-10^+^ Tregs from healthy donor PBMCs *in vitro.* Moreover, *in vivo* monoclonization of germ free (GF) mice with *P. distasonis* significantly increased the amount of IL-10^+^ CD4^+^ T cells in the spleen and mesenteric lymph nodes (94). In two distinct models of EAE, Berer et al. found that GF mice colonized with fecal material from MS-affected twins exhibited increased incidence and severity of disease compared to mice colonized with fecal material from non-MS twins. The relative protection afforded by the non-MS twin FMT was abrogated by administration of an anti-IL-10 neutralizing antibody, indicating that the FMT influenced CNS autoimmunity in an IL-10 dependent manner. Moreover, the mice given the MS FMT had a marked absence of IL-10^+^ Treg induction in the mesenteric lymph nodes (95).

Several studies have indicated that both the prophylactic and therapeutic administration of probiotics can reduce the severity of MOG_35-55_ and PLP_139-151_ EAE (104–106). Probiotic treatment was shown to suppress Th17 cell differentiation, promote the expansion of IL-10 producing T cells in the mesenteric lymph nodes and the CNS, and increase systemic IL-10 levels in serum (104). Two separate MS patient studies have also shown that administration of probiotics can increase the relative frequency of IL-10^+^ Tregs and levels of IL-10 in serum from the blood (107, 108). Furthermore, administration of a probiotic containing *Lactobacillus*, *Bifidobacterium* and *Streptococcus* was found to increase the gene expression of *IL-10RA* on monocytes derived from MS patient PBMCs (108).

### 3.2 Diet

The human diet plays a key role in influencing the gut microbiome, thus identifying dietary factors that lie upstream of the microbiome provides insight into potential therapeutic interventions for MS patients. A link between diet and autoimmune neuroinflammation has been demonstrated. For example, a cellulose rich diet which promotes the accumulation of *Lactobacillaceae* in the intestine, alleviates EAE in conjunction with an increase in IL-10^+^ CD4^+^ T cells (109). The amino acid tryptophan, which is obtained through our diet, can induce regulatory IL-10 producing T cells both *in vitro* and *in vivo* during EAE (110, 111).

Short-chain fatty acids (SCFAs) including acetate, butyrate, and proprionate are produced by the colon during the bacterial fermentation of dietary fibers. Progressive MS patients have been shown to have lower levels of SCFAs in the blood. Of note, oral SCFA administration to mice increases the number of IL-10^+^ T cells in the CNS during EAE (112). Furthermore, SCFA treated glial cells induce the production of IL-10 by T cells *in vitro* (112). In the context of EAE, administration of the SCFA propionate exands CNS-resident Tregs in an IL-10R dependent mechanism that further leads to an increase in IL-10 production by Tregs (113). In MS, increases in Enterobacteriaceae have been shown to be accompanied by reduced SCFA levels, and these alterations were more pronounced in patients with a higher burden of disease (114).

Other dietary modulations have been shown to improve MS and EAE, although a direct link to IL10R signaling was not investigated. In a small cohort of MS patients, a high-vegetable/low-protein diet increased the abundance of fecal *Lachnospiraceae* that correlated with a decrease in IL-17^+^ CD4^+^ T cells and an increase in IL-10^+^CD14^+^ monocytes in the blood as well as a reduction in relapse rate compared to “western diet” MS patients (115). Administration of a nutritional supplementation of non-fermentable fiber in early adult life, which promotes increases in *Helicobacter, Enterococcus, Desulfovibrio, Parabacteroides, Pseudoflavonifractor and Osillibacter* and the production of cecal long chain fatty acids was shown to reduce the incidence of spontaneous EAE. Authors did not specifically report on IL-10 production but did observe an increase in T cell-derived IL-4 and IL-5 (116). Moreover, an isoflavone diet has also been shown to protect against EAE, and the isoflavone-free diet promoted a microbiome that was more reminiscent of an MS microbiome (117). Dietary guar gum has also been shown to attenuate EAE, notably independent of SCFA, and these beneficial effects are primarily due to reduced T cell priming and migration to the CNS (118). Lastly, intermittent fasting in the context of EAE increased the abundance of *Lactobacillaceae*, *Bacteroidaceae*, and *Prevotellaceae* in conjunction with a reduction in intestinal IL-17-producing T cells and improved EAE outcomes (119). In these dietary modulations, it will be of interest to examine their impact on IL-10 production and IL-10R signaling.

### 3.3 Vitamin D

During the cooler months, latitude is the strongest determinant for the amount of vitamin D produced by UVB radiation, thus the amount of vitamin D absorbed by skin drastically decreases as latitude increases (120). A meta-analysis published in 2011 found a significant increase in MS prevalence at higher latitudes (121). Similarly, individuals with a genetic predisposition to vitamin D deficiency are at higher risk of developing MS (122, 123). The expression of the MS risk gene HLA-DRB1*1501 is regulated by a vitamin D responsive promoter (124), and the level of serum 1,25(OH)_2_D_3_ in MS patients is inversely correlated with disease progression (125, 126), new CNS lesion formation (127), and risk of relapse (128, 129).

There are direct impacts of Vitamin D on the IL-10/IL-10R axis in the context of MS and EAE. Vitamin D supplementation trials in MS patient cohorts have shown that high dose vitamin D elevates the proportion of IL-10 producing CD4^+^ T cells (130), increases levels of cell proliferation (131), and leads to a global increase in IL-10 levels in the serum of relapsing remitting MS patients (132–134). However, the effect of 1,25(OH)_2_D_3_ on T cells may be indirectly linked to its influence on other immune cell subsets as since 1,25(OH)_2_D_3_ was found to dampen the differentiation and maturation of APCs resulting in increased production of IL-10 concomitant with a reduced generation of alloreactive T cells (135, 136). Although less studied in the context of CNS autoimmunity, 1,25(OH)_2_D_3_ also influences B cells and has been linked to enhanced IL-10 production by activated human B cells (137).

In the context of EAE, continual administration of 1,25(OH)_2_D_3_ inhibits clinical disease in both prophylactic and therapeutic modalities (138–141). Similar to *in vitro* experiments, vitamin D3 supplementation led to an increased production of IL-10 by spleen and lymph node CD4^+^ T cells and a skew towards Treg and Th2 phenotypes (140). Furthermore, adoptive transfer of DCs cultured with 1,25(OH)_2_D_3_ into EAE mice dampened disease severity, inhibited the infiltration of Th1/Th17 immune cells into the CNS while increasing the representation of IL-10^+^ CD4^+^ T cells (142). A causal relationship between vitamin D and the IL-10/IL-10R axis has been demonstrated: Specifically, unlike IL-10 sufficient littermates, vitamin D supplementation protects neither IL-10 nor IL-10Rb deficient mice from developing severe EAE. Since reciprocal bone marrow chimera experiments revealed that IL-10 derived from both the radiosensitive and radioresistant cell compartments was necessary for protection against EAE, the precise IL-10 producing cell type in this study remains unidentified, and the nature of the IL-10 receiving cell type was not determined (141). Further research into IL-10 sensing cells following vitamin D supplementation will be an important next step in elucidating its benefits for MS patients.

In summary, Vitamin D has a direct impact on the IL-10/IL-10R axis in MS and EAE. Vitamin D may also have an indirect impact on the IL-10/IL-10R axis via the microbiome (143), which in turn can impact neuroinflammation, however this is not well-studied.

### 3.4 EBV

Epstein-Barr Virus (EBV) is a common human gammaherpesvirus that persists in more than 90% of the population worldwide (144). Recently, a longitudinal analysis provided strong causal evidence that EBV infection is a necessary co-factor for the development of MS (145). Previous and ongoing research has led to the development of several hypotheses on how EBV confers a greater risk of MS susceptibility including molecular mimicry and the generation of pro-encephalitogenic B cells (146). Interestingly, EBV encodes a viral homolog of IL-10 (vIL-10) (also known as BCRF1), which has approximately 80% structural similarity to its human equivalent (147). However, vIL-10 acts as a selective agonist that binds with lower affinity to the IL-10R (148). Despite binding to the IL-10R, vIL-10 does not influence DC functioning to the same extent as endogenous IL-10 – it is a poor inducer of STAT3 phosphorylation and is less effective at dampening the production of pro-inflammatory cytokines following LPS treatment (149). In agreement, Jog et al. found that vIL-10 binding to IL-10R interfered with hIL-10 induced STAT3 phosphorylation, thus indirectly inhibiting the ability for IL-10 to induce anti-inflammatory cytokine production (150). Although the existing literature on vIL-10 and MS is limited, we do know that vIL-10 protects EBV infected B cells against detection and elimination by dampening the secretion of antiviral cytokines and by preventing NK cell mediated killing (151). This may allow for pro-inflammatory EBV^+^ B cells to persist and contribute to CNS autoimmunity. Exploring how vIL-10 influences B cell populations during MS may allow us to further understand how EBV influences MS disease.

## 4 B cells as a bridge between the environment and IL-10R signaling in MS/EAE

Since the first description of IL-10^+^ B cells, a heterogenous collection of Bregs have been described in various disease contexts, and the specific signals controlling their development- several which can be influenced by the environment, are now of significant interest (152). For example, in rheumatoid arthritis products from the gut microbiota drive the production of IL-1β and IL-6 which in turn promotes the differentiation of IL-10^+^ Bregs (153). Butyrate supplementation, a metabolite produced by the microbiota, promotes an increased frequency of IL-10^+^ Bregs in rheumatoid arthritis patients, and mice lacking IL-10 producing B cells do not experience the same disease suppression following butyrate treatment (154). Other studies have shown similar expansions of IL-10^+^ B cells in response to other microbiota-derived metabolites including acetate and pentanoate (155, 156), indicating the role of microbial communities in shaping IL-10 levels by modulating the B cell population.

Following B cell receptor engagement, B cells develop into plasma cells whose “day job” is to produce antibodies to protect the host against re-infection. However, plasma cells can also provide important regulatory functions, even at distal locations, through production of anti-inflammatory molecules such as IL-10. In the context of the CNS, complementary mouse and human studies have verified that gut-derived IgA^+^ plasma cells can migrate to the brain meninges at homeostasis (157). Studies in MS (158) and EAE (38) also detected microbiota-reactive IgA^+^ plasma cells originating from the gut in the inflamed CNS, and adoptive transfer of IgA^+^ plasma cells isolated from the small intestine can reduce EAE severity in an IL-10 dependent manner.

**Figure 2:**
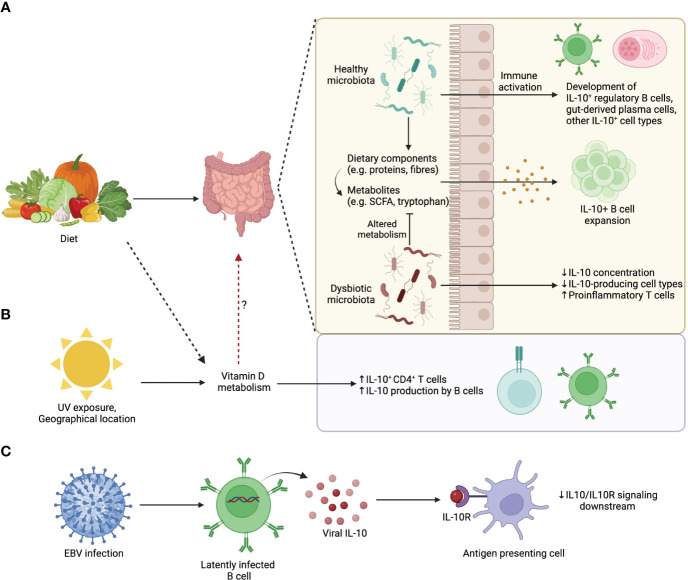
Environmental influences on IL-10-producing B cells. **(A)** Dietary patterns influence gut microbiota composition. A healthy microbiota and/or its associated diet-derived metabolites can promote the development of IL-10 producing commensal-reactive B cells and IgA+ plasma cells. **(B)** UV exposure from sunlight, which is influenced by geographical location, changes Vitamin D availability that upon metabolism promotes IL-10 production by lymphocytes. **(C)** EBV latently infected B cells produce viral homologs of IL-10 that compete with endogenous IL-10 for IL10R binding. Viral IL-10 homologues are less efficient than endogenous IL-10 at triggering downstream signaling.

Of note, deletion of IL-10 production specifically in plasma cells results in exacerbated EAE, and adoptive transfer of IL-10 competent plasma cells into IL-10^-/-^ EAE mice is sufficient to attenuate disease (38). This means that plasma cell derived IL-10 is both necessary and sufficient to dampen EAE, although it is highly likely that other IL-10 producing cells amplify these initial regulatory steps. With these data in mind, we propose a model whereby environmental factors operate through the gut microbiota promote IL-10^+^ B cell populations with the capacity to directly or indirectly regulate CNS autoimmunity during MS and EAE (**Figure 2**). It is likely that the recipients of IL-10 in this model are IL-10Rα expressing regulatory immune cell populations such as microglia, DCs, and regulatory T cell subsets. In support of this, there is evidence that IL-10^+^ Bregs are important for the differentiation of Tregs during EAE (75, 159, 160).

## 5 Conclusions

While it has been shown that lower IL-10 levels have a negative impact on MS/EAE, and that environmental exposures impact IL-10 levels, our understanding of what IL-10R expressing cell types(s) respond to IL-10 to alter neuroinflammation, and the environmental factors that impact IL-10R signals, is less comprehensive. In this vein, it is critical to not only understand the specific environmental contexts that influence IL-10 production, but also what cell types receive IL-10. The conflicting evidence between IL-10 knockout studies and cell specific IL-10R knockout studies in EAE indicate that there is more to be understood about IL-10R signaling during CNS autoimmunity. Exploring how these IL-10R-expressing cell types respond to environmental stimuli reframes the focus from the IL-10 producing cell type(s) to the cell-specific downstream effects of IL-10R signaling, and how environmental factors impacts these signals. Furthermore, we propose that B cells are the critical link between environmental stimuli and IL-10R signaling during MS/EAE. Identifying environmental factors that modulate the IL-10/IL-10R axis has the potential to provide new insights into therapeutic intervention for MS patients.

## Author contributions

EB wrote the article with editorial oversight from AW and JG. All authors contributed to the article and approved the submitted version.
